# Assessing sleep using the Pittsburgh Sleep Quality Index (PSQI) among comorbid HIV and psychiatric outpatients

**DOI:** 10.4102/sajpsychiatry.v31i0.2366

**Published:** 2025-04-09

**Authors:** Ahmad Peerbhay, Prinesh Miseer, Karishma Lowton

**Affiliations:** 1 Department of Psychiatry, Faculty of Health Sciences, University of the Witwatersrand, Johannesburg, South Africa

**Keywords:** HIV, PLWH, psychiatric disorders, psychiatric outpatients, sleep quality, sleep disorders, Pittsburgh Sleep Quality Index

## Abstract

**Background:**

Sleep is an essential component of physical and mental health. HIV and mental illness are both risk factors for developing sleep problems. There is a paucity of sleep research in a population with comorbid HIV and mental illness.

**Aim:**

This research study aimed to determine the prevalence of sleep disturbances among comorbid HIV and psychiatric outpatients using the Pittsburgh Sleep Quality Index (PSQI). A further aim was to identify socio-demographic and clinical variables that may significantly correlate with results of their PSQI scores.

**Setting:**

Luthando Neuropsychiatric Clinic at Chris Hani Baragwanath Academic Hospital in Johannesburg.

**Methods:**

This was a cross-sectional study assessing the sleep of comorbid HIV and psychiatric outpatients using the PSQI. A global score of 5 or greater is indicative of poor sleep quality. Eligible participants completed the self-administered PSQI, and socio-demographic and clinical data were obtained from their records.

**Results:**

A total of 71.6% of participants had an overall PSQI score of ≥ 5, indicating poor sleep quality. HIV-related factors were not predictors of poor sleep outcomes. Female gender, unemployment, absence of alcohol use and selective psychiatric diagnoses were associated with poor sleep quality. None of the participants had a sleep disorder formally diagnosed.

**Conclusion:**

Sleep disturbances are highly prevalent in patients with comorbid HIV and mental illness. Actively screening this population, training of psychiatrists in diagnosing sleep disturbances and interventions to improve the quality of sleep are needed.

**Contribution:**

This research highlights the prevalence of sleep disturbances in patients with HIV and mental illness.

## Introduction

Sleep is a vital, restorative physiological process.^1^ Insufficient sleep is a cause of and symptom of adverse medical and mental illnesses, and is a public health concern.^2^ People with sleep problems report poorer health, deficits in energy, cognitive functioning and reduced quality of life.^3^ This results in increased visits to health professionals, use of prescription and over-the-counter drugs.^3,4^ Furthermore, poor sleep quality and decreased total sleep time are linked to a greater risk for morbidity and mortality.^1^

Sleep disturbances interact with common mental disorders in a bi-directional manner.^5^ The most common sleep disturbance observed in psychiatric patients is insomnia.^6^ Results of a meta-analysis described that psychiatric patients had significantly reduced sleep efficiency and total sleep time. This was accounted for by decrements in non-rapid eye movement (NREM) sleep as measured by polysomnography.^7^ In a study of forensic psychiatric patients, 30% had a sleep disorder and 49.1% reported poor sleep quality.^8^ Berlin et al. found that 80 out of 100 psychiatric outpatients had a sleep disorder and 72 of those had insomnia.^9^ A more recent and local study conducted on 90 psychiatric outpatients found 50% had poor sleep quality.^10^ Untreated insomnia is associated with increased suicidal ideation, poor treatment response and may increase the risk of developing depression by two-fold.^6,11^ In clinical practice, many sleep disturbances are usually misconstrued as part of the psychiatric condition and not treated appropriately.^12^

Results of a meta-analysis described a 58% prevalence of sleep disturbances in people living with HIV (PLWH).^13^ The pathology of sleep disturbances in HIV is not well understood. It has been postulated that HIV can negatively affect normal sleep via two main mechanisms. The mechanisms are direct effects of the immunological response to the virus, and indirectly through the neurotoxic effects of the immunopathological response in the central nervous system. During the early stage, acute immunological response to the virus results in longer sleep duration but with more fragmented sleep and daytime fatigue. Following seroconversion, persistent expression of modulatory cytokines because of immune activation, disrupts normal sleep architecture.^14^ Encephalogram readings of PLWH revealed longer sleep-onset latency and a shorter total sleep time.^15^ Variables that mediate sleep in PLWH include, antiretroviral therapy (ART) regimen, time since HIV diagnosis, viral load, CD4 count and psychosocial stressors.^16,17,18^ HIV positive individuals with sleep problems are more likely to be non-adherent to their treatment resulting in disease progression, the development of resistant strains and consequently treatment failure.^19,20^

The association between CD4 counts and sleep remains unclear. Seay et al. found greater sleep disturbances were associated with lower CD4 counts.^21^ However, a South African cohort study established that poor sleep quality was associated with higher CD4 counts, when adjusting for depression severity, daytime sleepiness and pain.^22^ With regard to ART, a longitudinal prospective study examined sleep quality of HIV positive patients pre- and 1 year post-ART initiation. Results described significantly poorer sleep quality on ART with a 15.5 times increased risk compared to pre-ART. The ART regimen included non-nucleoside reverse transcriptase inhibitor (NNRTI) nevirapine or efavirenz.^18^ Elevated plasma levels of efavirenz correlated with poor sleep disturbances in HIV.^23^ Dolutegravir (DTG), an integrase strand transfer inhibitor (INSTI), which has replaced efavirenz as a first-line ART in South Africa, has also been linked to sleep problems. Research suggests that DTG is associated with mild neuropsychiatric manifestations, the most common being insomnia and sleep disorders.^24^ These results, however, did not display a statistical significance.

Sleep disturbances can be evaluated with specialised equipment such as actigraphy and polysomnography. Polysomnography remains the gold standard measurement of objective sleep characteristics.^25^ However, it can be costly, time-consuming and not readily available in most centres. Sleep analysis is limited to one or two nights, leading to a limited evaluation of sleep patterns. Furthermore, a laboratory setting can impact the sleep pattern because of environmental changes.^17^ Sleep questionnaires have been suggested as screening instruments in clinical practice due to its ability to measure subjective sleep quality and the cost benefit compared to polysomnography.^17,26^

Thus far, studies have individually investigated sleep disturbances among PLWH and psychiatric patients. Given the prevalence of sleep disturbances in both populations, there is a paucity of sleep research with comorbid HIV and mental illness. Our aim was to explore sleep quality using the Pittsburgh Sleep Quality Index (PSQI) and associated predictors among comorbid HIV and psychiatric outpatients.

## Research methods and design

### Study design and setting

This study was a cross-sectional assessment of the overall sleep quality, and its seven components, in adult outpatients attending Luthando Neuropsychiatric Clinic at Chris Hani Baragwanath Academic Hospital (CHBAH). It is a specialised dual diagnosis clinic, treating PLWH and comorbid mental illness.

### Inclusion and exclusion criteria

#### Inclusion criteria

- Participants older than 18 years of age
- HIV positive and on ART
- Diagnosed with a psychiatric condition
- Patients who were stable after clinic review
- Have a basic understanding of the English language

#### Exclusion criteria

- Outpatients who had a major neurocognitive disorder that would affect understanding and completion of the questionnaire
- Outpatients who are severely or acutely ill, impacting on consent and completion of the questionnaire

### Data collection and instrument (Pittsburgh Sleep Quality Index)

The PSQI is a self-reported questionnaire developed by Buysse et al. which assesses sleep quality over the past month. It is the most widely used scale to assess sleep and has been used in a range of clinical and non-clinical studies.^27^ Nineteen individual items generate seven component scores: subjective sleep quality, sleep latency, sleep duration, sleep efficiency, sleep disturbances, use of sleeping medication, and daytime dysfunction. A PSQI score ≥ 5 carries a diagnostic sensitivity of 89.6% and specificity of 86.5% in distinguishing poor from good sleepers.^26^ A systematic review of 37 studies demonstrated that the PSQI has strong reliability and validity.^28^

Information regarding the study was explained to participants via poster presentations, in addition to an information sheet. Adults were invited to participate after clinical review by the doctor. Participants completed their demographic information and the self-administered PSQI. Clinical information included CD4 count, viral load, psychiatric diagnosis, ART regimen, duration of illness and current substance use. These data were obtained from the clinic file and recorded on the data collection sheet by the principal investigator. A study number was assigned to the data sheet and the corresponding PSQI to ensure confidentiality in the study. The sample size was calculated using the formula: *n* = *Z*^2^*P(1-*P*)/*d*^2^, where *n* = sample size, *Z* = *Z* statistic for a level of confidence (1.96 for 95% confidence level), *P* = Expected prevalence or proportion and *d* = Precision. Based on a prevalence of 58% sleep disturbances among PLWH^13^ in the literature, a sample size of 222 patients would be adequate at a precision of 6.5%. A total of 257 individuals provided informed consent and were enrolled in the study during July–December 2023.

### Data analysis

The data were captured in Microsoft Excel^TM^. All statistical analyses were conducted using R software (R version 4.0.1; https://www.r-project.org). The data were evaluated for normality using Shapiro–Wilk tests, which revealed no deviations from normality. Subsequently, the data were analysed using suitable parametric or non-parametric tests, as appropriate. Analyses were two-tailed, and model-level significance was set at 0.05. Continuous data were reported as mean and standard deviation and categorical data were reported as counts and percentages and presented in charts, tables or text.

To assess the sleep quality of the sample population, the proportion of patients with scores < 5 (good sleep quality) was compared to those with scores of ≥ 5 (poor sleep quality) using a chi-squared contingency table analysis to assess whether the proportion of patients sampled with sleep disorder was greater or less than chance. The prevalence of poor sleep quality was reported as a percentage of the total sample size with 95% CIs. A frequency distribution plot of the total scores was also generated. The distribution of scores (from 0 to 3) for each of the seven component categories was analysed using a chi-squared contingency tests.

Fisher’s exact tests were used to analyse whether gender, employment status and substance use predicted sleep quality. Unadjusted odds ratios were provided for significant outcomes. The categorical socio-demographic and clinical predictors of sleep disturbances among comorbid HIV and psychiatric patients were analysed using Pearson’s chi-squared goodness of fit tests (followed by binary post hoc tests for significant outcomes). The influence of continuous variables on sleep quality was analysed using Welch’s *t*-tests (age, years since the HIV diagnosis) or Mann–Whitney *U* tests (CD4 count and viral load).

### Ethical considerations

Permission to conduct the study was obtained from the relevant hospital authorities. Ethical clearance to conduct this study was obtained from the University of the Witwatersrand Human Research Ethics Committee (Medical) (No. M230445). Permission was obtained from the Sleep Medicine Institute of the University of Pittsburgh for use of the PSQI for educational and non-commercial purposes. Participants’ confidentiality was strictly maintained. No information obtained during this study was used for purposes other than fulfilling the study objectives.

## Results

The data set comprised of a total of 257 patients at Luthando Neuropsychiatric Clinic at CHBAH.

### Socio-demographic data

Patients in the study had a mean age of 47.30 years (Table 1). Significantly more patients were female (79%). Almost all patients were African (99%). Significantly, more patients were single (71%), finished secondary school (67%) and were unemployed (86%).

**TABLE 1:** Socio-demographic data collected from patients at the Luthando Neuropsychiatric Clinic at CHBAH.

Variables	Values (% of total number of study population)		Poor sleep quality		Good sleep quality	
	*n*	%	*n*	%	*n*	%
Age (years)	47.30	9.33	-	-	-	-
**Gender**						
Female	203	79	153	75	50	25
Male	54	21	35	65	19	35
**Race**						
African	254	99	195	77	59	23
Mixed	3	1	3	100	-	-
**Marital status**						
In relationship	39	15	32	82	7	18
Divorced	18	7	17	94	1	6
Single	182	71	136	75	45	25
Widowed	18	7	13	72	5	28
**Highest level of education**						
Primary	37	15	29	78	8	22
Secondary	173	67	135	78	38	22
Tertiary	47	18	34	72	13	28
**Employment status**						
Employed	35	14	19	54	16	46
Unemployed	222	86	165	74	57	26

Age is shown as a mean (s.d.) and other variables are shown as a count (percentage) and categorised according to poor versus good sleep quality (*N* = 257).

### Clinical data

A significant number of patients reported not using substances (88%; Table 2). Of the 12% who did, significantly more used alcohol (11%) than cannabis (1%). A significant majority of patients were on an INSTI-based ART regimen (82%; Table 2). Patients had 1, 2 or 3 of seven psychiatric diagnoses. For the analyses, the total number of patients for each diagnosis was calculated, resulting in a total of 362 records. Major depressive disorder and bipolar disorders were grouped as primary mood disorders whereas schizophrenia and schizoaffective disorders were grouped as primary psychotic disorders. A significantly greater proportion of the patients had a psychotic disorder secondary to HIV, followed by a primary mood disorder and a mood disorder secondary to HIV (Table 2).

**TABLE 2:** Clinical variables collected from patients at the Luthando Neuropsychiatric Clinic at CHBAH.

Variables	Values (% of total number of study population)		Poor sleep quality		Good sleep quality	
	*n*	%	*n*	%	*n*	%
**Substance use (excluding tobacco or tobacco products)**						
Alcohol	28.00	11.00	15	54.00	13	46.00
Cannabis	3.00	1.00	2	67.00	1	33.00
Nil	226.00	88.00	167	74.00	59	26.00
**ART regimen**						
Integrase strand transfer inhibitor	210.00	82.00	165	79.00	45	21.00
NNRTI	17.00	6.00	9	53.00	8	47.00
Protease inhibitor	30.00	12.00	24	80.00	6	20.00
**Psychiatric diagnoses**						
Borderline personality disorder	16.00	4.00	15	94.00	1	6.00
Mood disorder secondary to HIV	79.00	22.00	49	62.00	30	38.00
Neurocognitive disorder secondary to HIV	36.00	10.00	27	75.00	9	25.00
Primary mood disorder	97.00	27.00	76	78.00	21	22.00
Primary psychotic disorder	22.00	6.00	15	68.00	7	32.00
Psychotic disorder secondary to HIV	103.00	28.50	66	64.00	37	36.00
PTSD	9.00	2.50	8	89.00	1	11.00
**Other clinical variables**						
CD4 count	635.67	301.82	-	-	-	-
Viral load	2945.25	16490.11	-	-	-	-
Years since HIV diagnosis	13.68	5.94	-	-	-	-

Data expressed as count (percentage) where *N* = 257 for all variables except psychiatric diagnoses (*N* = 362). Variables are shown as a count (percentage) and categorised according to poor versus good sleep quality. Other clinical variables are shown as a mean (s.d.).

ART, antiretroviral therapy; NNRTI, non-nucleoside reverse transcriptase inhibitor; PTSD, Post-traumatic stress disorder.

### Sleep quality

**FIGURE 1:**
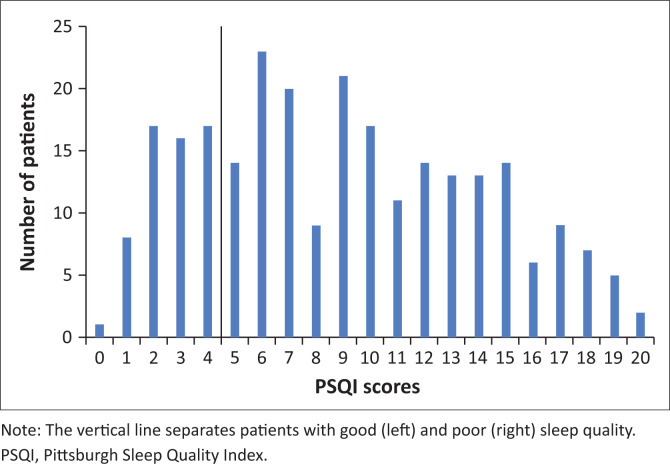
Frequency distribution of patients by Pittsburgh Sleep Quality Index score in patients at the (Luthando Neuropsychiatric Clinic at CHBAH).

Sleep quality was categorised as good or poor based on the total PSQI. The prevalence of sleep disturbances was 71.6% (95% CI: 66% – 77%). A frequency plot of the total PSQI scores (Figure 1) showed a skewed distribution in favour of the numbers of patients with poor quality because the cut off for good sleep was < 5.

Table 3 shows the scores for each of the seven PSQI components. Each of the seven subscales of the PSQI were rated from 0 to 3 with zero being no issues and three representing severe difficulties.

**TABLE 3:** The counts (percentage) of scores from 0 to 3 for each of seven Pittsburgh Sleep Quality Index components collected from patients at the (Luthando Neuropsychiatric Clinic at CHBAH), (*N* = 257).

Score	Components													
	Subjective sleep quality		Sleep latency		Sleep duration		Sleep efficiency		Sleep disturbance		Use of sleep medication		Daytime dysfunction	
	*n*	%	*n*	%	*n*	%	*n*	%	*n*	%	*n*	%	*n*	%
0	35	14	83	32	126	49	146	57	17	7	120	47	95	37
1	64	24	87	34	51	20	35	14	145	56	16	6	76	30
2	58	23	40	16	26	10	21	8	83	32	14	5	54	21
3	100	39	47	18	54	21	55	21	12	5	107	42	14	12

Statistically significant (*p*-values < 0.05) components of the PSQI included: sleep quality, sleep latency, sleep duration, sleep efficiency, sleep disturbance, sleep medication and daytime dysfunction.

### Sleep quality versus socio-demographic and clinical variables

Comparisons were made between the sleep quality and socio-demographic factors. The age of patients with good sleep quality (mean = 46.32, s.d. = 10.20 years) did not differ significantly from those with poor quality (mean = 37.68, s.d. = 8.96 years) (Welch’s *t*-test = –1.00, *df* = 118.46, *p* = 0.320).

**FIGURE 2:**
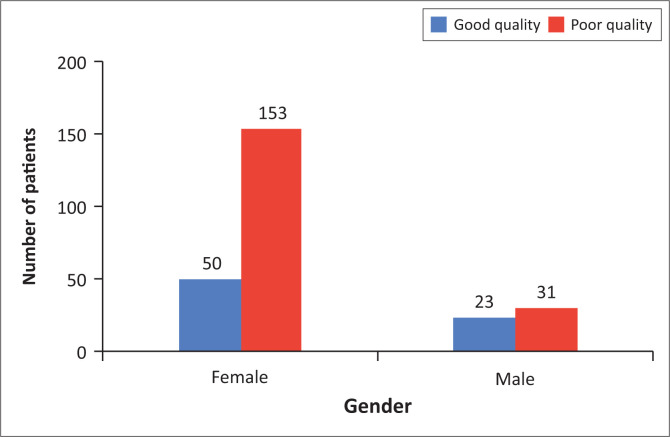
The sleep quality of female and male patients at the (Luthando Neuropsychiatric Clinic at CHBAH).

There were significant gender differences in sleep quality (Fisher’s exact test: *p* = 0.017). For females, 25% reported good sleep quality and 75% reported poor sleep quality, whereas 43% males reported good sleep quality and 57% reported poor sleep quality (Figure 2). Females were 2.27 times (OR = 2.27, 95% CI: 1.21–4.25) more likely than males to suffer from poor sleep quality.

**FIGURE 3:**
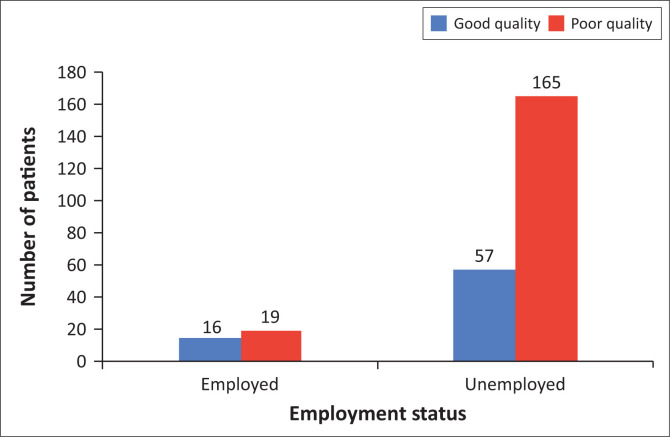
The sleep quality of employed and unemployed patients at the (Luthando Neuropsychiatric Clinic at CHBAH).

Employment status did predict sleep quality (Fisher’s exact test: *p* = 0.025). Unemployed patients (74%) had poor sleep quality compared to those who had good sleep quality (26%) (Figure 3). In contrast, a similar proportion of employed patients had poor (54%) and good sleep (46%) quality. Unemployed patients were 2.44 times (OR = 2.44, 95% CI: 1.17–5.06) more likely than employed to suffer from poor sleep quality. Marital status (χ^2^ = 5.13, *df* = 3, *p* = 0.162) and highest level of education (χ^2^ = 0.11, *df* = 2, *p* = 0.945) were not significant predictors of sleep quality. Race was not analysed because almost all patients were African.

Cannabis users were excluded from the analyses because of the small sample size (*N* = 3). Participants not using substances and participants using only alcohol were analysed. The absence of substance use did predict poor sleep quality (Fisher’s exact test: *p* = 0.043). Of the population not taking a substance, 74% had poor sleep quality. In addition, of the population of patients who used alcohol, 54% had poor sleep quality and 46% had good sleep quality. Patients not using alcohol were 2.45 times (OR = 2.45, 95% CI: 1.10–5.46) more likely to suffer from poor sleep quality.

**FIGURE 4:**
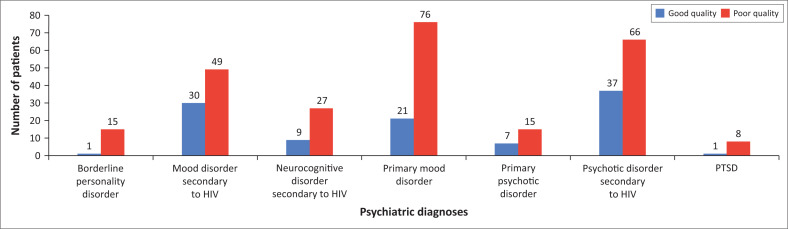
The sleep quality of patients in relation to seven psychiatric diagnoses at the (Luthando Neuropsychiatric Clinic at CHBAH).

The psychiatric diagnosis of patients was significantly associated with poor sleep quality (χ^2^= 13.73. *df* = 6, *p* = 0.033). A greater proportion of patients had poor sleep quality than good sleep quality for six diagnoses, that is, borderline personality disorder, mood disorder secondary to HIV, mild neurocognitive disorder secondary to HIV, primary mood disorder, psychotic disorder secondary to HIV and PTSD (Figure 4). The exception was patients with primary psychotic disorder which did not differ in sleep quality (Figure 4).

A total of 142 patients had only one psychiatric diagnosis. Of these, 37 (32%) had good sleep quality and 78 (69%) had poor sleep quality. The remaining 115 patients had multiple diagnoses, of which 36 (32%) had good sleep quality and 106 (68%) had poor sleep quality. The difference between the one versus multiple diagnoses regarding poor sleep quality was not statistically significant (Fisher’s exact test: *p* = 0.266).

None of the other clinical factors were significant predictors sleep quality, including years since HIV diagnosis (*t* = -0.22, *df* = 127.17, *p* = 0.825), CD 4 count (*W* = 6131.5, *p* = 0.277), viral load (*W* = 7225, *p* = 0.225) and ART regimen (χ^2^ = 3.12, *df* = 2, *p* = 0.210).

## Discussion

To our knowledge, this is the first study to examine sleep quality in patients with comorbid HIV and mental illness. The key findings included 71.6% of participants reporting poor sleep quality. This was higher compared to a meta-analysis describing a prevalence (58%) of self-reported sleep disturbances in PLWH without diagnosed mental illness.^13^

Stigma, employment discrimination and higher levels of unemployment have been independently documented in studies of psychiatric patients and studies of PLWH.^29,30^ Furthermore, studies have highlighted the deleterious impact of psychosocial factors such as unemployment on sleep.^31,32,33^ Compared to these studies, our findings support the higher prevalence of sleep disturbances found in the unemployed population.

Sleep problems in HIV-positive females are more common compared to males.^13,34^ Females are more prone to sleep disturbances because of higher rates of mental illness such as depression and anxiety.^35^ This correlated with our findings even when considering the representation of gender in this study population. Moreover, peri and postmenopausal women are specifically more affected as compared to premenopausal women.^13,36^ This could account for the higher prevalence in our sample as our average age of female participants was 47 years.

Previously, sleep disorders in HIV were largely attributed to immunosuppression. However, sleep disturbances have been linked more to psychological distress than immune status from ART and viral control.^34^ Our findings mirrored descriptions of previous research as clinical variables such as viral load, CD4 count, and HIV duration were not significant predictors of poor sleep.^34,37,38^

Neuropsychiatric adverse effects such as, insomnia and sleep disorders because of DTG, have been described in the literature.^24^ However, recent studies examining sleep quality using the PSQI found an insignificant association with DTG.^39,40^ Our findings were consistent with these studies, as there were no significant associations between DTG and sleep quality.

Poor sleep has been documented in patients with mood disorders.^5,41^ This was supported by Wibbler et al., who found that mood symptoms were an important factor contributing to sleep disturbances in PLWH.^38^ This correlated with our study whereby 78% of those diagnosed with a primary mood disorder, reported poor sleep quality. Studies on sleep disturbances in borderline personality disorder patients displayed inconsistencies with prevalence and sleep architecture results.^42^ This statistically significant finding related to borderline personality disorder in our study, could be due to 100% of these participants having comorbid HIV and depression.

In our study population, there were no statistically significant differences between poor and good quality sleep for patients with a primary psychotic disorder. However, patients diagnosed with a psychotic disorder secondary to HIV had significantly poorer sleep quality on PSQI scores. This could be hypothesised by different pathological mechanisms between these two conditions.^43^ This further highlights comorbid HIV and mental illness effect on sleep quality and warrants further research in this population.

Post-traumatic stress disorder was also another predictor of poor sleep quality in our study population. Rates of PTSD are higher in PLWH than the general population and hyperarousal symptoms have been linked to sleep disturbances, which may explain this finding.^44^ There is a paucity of sleep research in this subset of patients and highlights the need for more evidence-based information.

Sleep disturbances are common in neurocognitive disorders and comorbid HIV. A study by Rubinstein et al. described 100% of their study population with both conditions having sleep disturbances.^45^ A high proportion of participants (75%) with a mild neurocognitive disorder secondary to HIV had poor sleep quality in our study population.

In our population, most participants reported sub-scale scores of 0 and 1 regarding sleep latency (66%), sleep duration (69%) and sleep efficiency (71%). This finding could be explained by the sedative properties of psychiatric medication prescribed. These medications have been associated with decreased sleep latency and increased sleep duration.^46^ Despite this, 62% of our study participants reported poor subjective sleep quality. The limited influence of psychiatric medication on sleep architecture may explain why patients reported overall poor sleep quality.^46^

Merrill et al. found that people with multiple psychiatric diagnoses had higher rates of insomnia and sleep apnoea compared to those with only one psychiatric diagnosis.^47^ In contrast, our study found similar rates of poor sleep in patients with either one psychiatric disorder (69%) or multiple psychiatric disorders (68%). The key difference between the study by Merrill et al. and our findings was that our study population focused on HIV-positive psychiatric patients. These findings may suggest that the presence of a comorbid HIV diagnosis impacts sleep quality in patients, regardless of the number of psychiatric conditions they have.

Study participants who reported alcohol use had better sleep quality. Acute alcohol use can act as a sedative by decreasing sleep latency; however, chronic use is associated with sleep disturbances.^48^ A smaller study that observed sleep quality in those with substance use and HIV found that alcohol and illicit drug use were not predictors of poor sleep, although the authors cautioned that it was likely due to a small study sample.^45^ We were unable to assess chronicity or a diagnosis of alcohol use disorder; therefore, future sleep research among patients with triple diagnoses: HIV, mental illness and a substance use disorder is warranted.

### Strengths and limitations

The strengths of our study included an adequate sample size and the use of a validated clinical tool to assess sleep disturbances. This is contributing to a growing area of research on sleep disturbances in populations with HIV and mental illness. The limitations of this questionnaire may include understanding of information required, language barriers and response bias. The cross-sectional design of the study and the methodology did not allow us to establish cause-and-effect correlations especially in high-risk populations like major neurocognitive disorders. Data on the psychotropic medication prescribed, which can affect sleep quality both positively and negatively, were not analysed. Lastly, no objective assessment, such as polysomnography, was conducted to assess sleep.

### Implications and recommendations

Healthcare workers should be cognisant of sleep issues and their relation to medical and mental illness. Psychiatry-trained doctors are required to diagnose and manage sleep disorders as per training regulations. The authors would like to highlight a possible gap in training and knowledge of these conditions as none of the participants had a sleep disorder formally diagnosed. Patients should be actively screened for any sleep difficulties and treated appropriately. Sleep hygiene and cognitive-behaviour therapy for insomnia (CBT-I) are effective non-pharmacological treatment options.^49^

## Conclusion

The results of this study indicate that poor sleep quality, as determined by the PSQI, is highly prevalent in patients with comorbid HIV and mental illness. Integrating assessment of sleep into routine care, providing a formal diagnosis where appropriate and instituting treatment would be effective in managing sleep-related issues in this population.
